# Recent Advances in Microbial Levan Production: Strategies for Yield Enhancement and Sustainability

**DOI:** 10.1155/ijfo/2705142

**Published:** 2026-06-22

**Authors:** Emmanuel N. Nkuruemma, Priscilla B. S. Albuquerque, Irapuan O. Pinheiro

**Affiliations:** ^1^ Institute of Biological Sciences, University of Pernambuco (UPE), Recife, Pernambuco, Brazil, upe.br

**Keywords:** downstream processing, fermentation optimization, levan production, levansucrase regulation, sustainability

## Abstract

Levan is a fructose‐based polysaccharide with prebiotic potential and expanding applications in the food, biomedical, and industrial sectors. However, its large‐scale production is limited by heterogeneous performance data, nonstandardized yield reporting, and suboptimal process design. This review presents an integrative bioengineering analysis of levan production by linking microbial diversity, levansucrase genetic regulation, enzyme functionality, and key process parameters. Based on 81 studies, substantial variability in levan production is observed, largely due to differences in cultivation strategies and analytical methods. Oxygen transfer limitations and substrate concentrations are identified as critical yet underexplored factors affecting productivity and scalability. Downstream processing challenges are also highlighted, particularly regarding their impact on product molecular weight, purity, and process sustainability. In addition, sustainability constraints are discussed in relation to energy and solvent‐intensive recovery operations. Overall, this review proposes a unified bioengineering framework to support the development of more scalable, standardized, and efficient levan production systems.

**Highlights**

• Levan production exhibits wide variability (<1 to >700 g L^−1^), largely influenced by microbial system selection and bioprocess design.
• High levan titers can be achieved under optimized conditions, reaching up to 387.4 g L^−1^ in batch systems and 746 g L^−1^ in continuous processes.
• Levan synthesis and polymer structure are strongly influenced by levansucrase regulation, enzyme localization, and metabolic context.
• Fermentation strategies (batch, fed‐batch, and continuous cultivation) determine key trade‐offs between productivity, substrate utilization, and process stability.
• The lack of standardized yield reporting (g g^−1^) remains a major limitation for cross‐study comparison and robust process evaluation.
• An integrative bioengineering framework is proposed to support rational strain selection, process optimization, and application‐driven levan production.

## 1. Introduction

**Figure 1:**
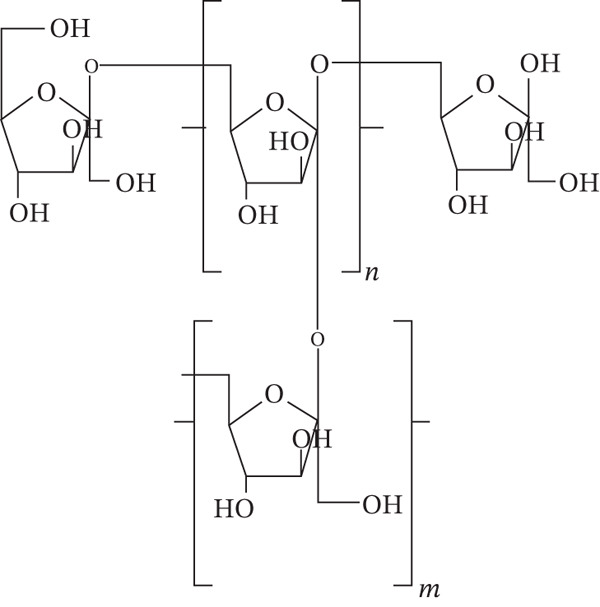
Structural representation of levan, a *β*‐(2 → 6)‐linked fructose polymer with *β*‐(2 → 1) branches, illustrating the repeating fructofuranosyl units and branched architecture characteristic of microbial levan.

The Sustainable Development Goals (SDGs) 2 and 3 emphasize the importance of ensuring access to sufficient food and optimal health for all [1]. Despite global efforts, foodborne diseases such as obesity, gastrointestinal disorders, diabetes, coronary heart disease, and cancer remain significant contributors to morbidity and mortality [2]. To address these health challenges, there has been growing interest in functional foods, particularly those containing prebiotics, such as levan, due to their potential health benefits [3]. Levan is a polysaccharide composed primarily of fructose molecules linked by a *β*‐(2 → 6) glycosidic bonds, with occasional *β*‐(2 → 1) branches [4] (Figure 1). As a member of the fructans family, which includes inulin and other dietary fibers [5], levan′s complex carbohydrate structure confers a range of functional properties [6]. These properties include film‐forming ability [7], antioxidant activity [6], anti‐inflammatory effects [8], biodegradability and nontoxicity [9, 10], rheological properties [11], water retention and immunomodulatory activity [4], emulsifying capacity and sweetening potential [4], and antimicrobial effects [12], making it valuable for applications in cosmetics, pharmaceuticals, food, and medicine [13]. Additionally, levan has been shown to be nonhemolytic and to exhibit antioxidant activity [14].

In recent years, interest in levan has expanded beyond its functional attributes to encompass its production pathways and the biochemical mechanisms underlying its biosynthesis. Levan can be synthesized via microbial fermentation using bacteria [15] and yeasts [16, 17], enzymatic conversion mediated by levansucrase [4], and, to a lesser extent, by certain plant species [18]. Among these approaches, microbial fermentation remains the most industrially relevant due to its scalability, controllability, and adaptability to process optimization. Moreover, microbially derived levan exhibits a wide range of physicochemical and biological properties, further reinforcing its industrial potential [4].

Importantly, the physicochemical and biological properties of levan are strongly influenced by the producing microorganism and biosynthetic conditions. Levan biosynthesis is primarily catalyzed by levansucrase (EC 2.4.1.10) [19], a GH68 fructosyltransferase, whereas other sucrose‐active enzymes, such as invertase (EC 3.2.1.26) may indirectly affect the process by competing for sucrose, thereby reducing substrate availability for levansucrase‐mediated polymerization [20].

However, despite significant advances, large‐scale levan production remains challenging. Key limitations include strain‐dependent variability, high substrate costs, and inefficiencies in downstream processing. In addition, process performance is highly sensitive to operational conditions. Parameters such as substrate concentration and associated osmotic stress [21], medium viscosity [22], and oxygen availability [23] play a critical role in levan synthesis. In particular, elevated sucrose concentrations and increased broth viscosity in high‐cell‐density systems can severely limit oxygen transfer [24].

Within this framework, the volumetric oxygen transfer coefficient (kLa) emerges as a key parameter governing oxygen mass transfer from the gas to the liquid phase, thereby directly impacting dissolved oxygen levels, cellular metabolism, and overall productivity. Additionally, dynamic changes in culture composition, including extracellular polymer accumulation and process additives, can further modulate kLa, influencing the oxygen uptake rate (OUR) and, ultimately, process efficiency [25].

Despite the growing body of literature on levan, several important gaps remain. Current studies often address microbial diversity, enzymatic mechanisms, or process optimization separately, with limited integration across these levels. Moreover, the lack of standardized performance metrics hampers reliable comparison between studies and limits the translation of laboratory findings to industrial applications.

In this context, the present review is aimed at: (i) comparatively analyzing levan‐producing microorganisms, with a focus on the relationship between strain diversity and levansucrase genetics and regulation; (ii) critically evaluating the impact of key fermentation parameters using normalized performance indicators where available; and (iii) examining downstream processing strategies in relation to product specifications and sustainability considerations.

Unlike previous studies, this review adopts an integrative bioengineering perspective by systematically linking genetic, enzymatic, process, and structural levels. Particular emphasis is placed on oxygen transfer limitations (kLa) and the lack of standardized evaluation criteria, two critical yet underexplored factors that constrain process optimization and scalable levan production.

## 2. Methodology

### 2.1. Literature Search Strategy

A systematic literature search was conducted to identify and analyze studies on microbial levan production, fermentation optimization, and downstream processing, as well as its industrial and biomedical applications. This review synthesizes data from 81 peer‐reviewed studies selected according to predefined criteria. The search was performed using PubMed, Google Scholar, and Web of Science, covering publications from 2001 to 2026. Keyword combinations included levan production, fermentation optimization, microbial strains, levansucrase, biosynthesis, and applications. Data were extracted and organized using Microsoft Excel and Word, whereas GraphPad Prism was used for data visualization. Figures and schematics were prepared using BioRender and Canva. References were managed using Zotero to ensure consistency and accuracy. Importantly, only experimentally reported values (e.g., levan concentration and productivity) were considered. Parameters such as yield coefficients (g g^−1^) were included only when explicitly reported by the original authors, and no estimations were performed to avoid overinterpretation.

### 2.2. Inclusion and Exclusion Criteria

Studies published between 2004 and 2026, written in English, and peer‐reviewed, were included. Eligible studies reported quantitative data on microbial levan production, including fermentation parameters, reactor configuration, and production metrics such as levan concentration, productivity, and, when available, yield coefficients (g g^−1^). Studies lacking quantitative experimental data, focusing exclusively on plant‐derived levan, or published as abstracts or nonpeer‐reviewed reports were excluded. Due to substantial heterogeneity in cultivation conditions, analytical methods, and downstream processing strategies, data were analyzed as reported ranges rather than statistically normalized values. Particular attention was given to process‐related factors affecting scalability, including oxygen transfer limitations, viscosity effects, and differences between shake‐flask and bioreactor systems.

## 3. Results and Analysis

### 3.1. Critical Analysis of Reported Levan Production Data

Recent studies have documented a wide diversity of microbial sources capable of producing levan, a fructan‐type exopolysaccharide predominantly synthesized in sucrose‐rich environments through the action of levansucrase, the key enzyme catalyzing the polymerization of fructose units [4, 12]. A comprehensive review of the current literature reveals that levan‐producing microorganisms, including *Bacillus subtilis, Bacillus velezensis, Bacillus amyloliquefaciens, Paenibacillus polymyxa, Azotobacter chroococcum, Gluconobacter japonicus, Brenneria goodwinii, Halomonas smyrnensis, Zymomonas mobilis, Leuconostoc citreum, and Leuconostoc mesenteroides*. In addition, yeast species such as *Saccharomyces cerevisiae, Pichia pastoris*, and *Suhomyces kilbournensis*, as well as certain acetic acid bacteria, have also been identified as efficient levan producers under suitable fermentation conditions [12, 13, 26].

In parallel, a wide array of substrates has been evaluated for levan production, with sucrose remaining the most commonly used and effective carbon source due to its strong induction of levansucrase activity [22, 26]. However, numerous studies have explored the use of alternative low‐cost feedstocks to enhance sustainability and reduce production costs. These include sugarcane molasses, mango peel hydrolysate, and sucrose‐enriched tomato juice, which have demonstrated promising potential for supporting microbial growth and levan synthesis [6, 12, 13]. Overall, the literature reflects increasing interest in optimizing both microbial strains and fermentation media, emphasizing the biotechnological versatility and industrial relevance of levan production. To provide a structured overview, Table 1 compiles reported levan production data across different microorganisms, substrates, and cultivation strategies. In addition to levan titers, the table includes key process parameters such as substrate concentration, fermentation mode, reactor configuration, and cultivation time, along with performance indicators including volumetric productivity and yield coefficients when available. This dataset enables a comparative assessment of current production strategies while highlighting existing limitations in data consistency and reporting.

**Table 1:** Comparative overview of microbial levan production systems, process conditions, and reported performance metrics.

Microorganism	Substrate	Substrate conc. (g L^−1^)	Process mode	Reactor type	Time (h)	Conc. (g L^−1^)	Average productivity (g L^−1^ h^−1^)	Yield (g/g) Yp/s	Analytical method	References
*Bacillus subtilis* MTCC441	Sugarcane Saccharum spp.	100	Batch	Flask (250 mL)	20	NR	NR	0.32	NMR and GPC	[27]
*Bacillus velezensis* KKSB6	Sucrose	200	Batch	Flask (600 mL)	72	187	2.60	0.93	HPLC	[19]
		800	Continuous	Flask (50 mL)	96	746	7.77	0.93		
		~300	Fed‐batch	Flask (50 mL)	72	183.5	2.48	0.55		

*Suhomyces kilbournensis*	Sugarcane molasses	400	Batch (SmF)	Flask (170 mL)	6	44.33	7.39	NR	FT‐IR	[12]
*Recombinant Pichia pastoris*	Sucrose	160	Batch	Bioreactor 1.5 L	59	72.9	1.24	0.45	HPLC	[28]
		12.83	Batch	Flask 250 mL	288	8.62	0.03	0.28		

*B. velezensis* SR1	Sucrose	50	Batch	Flask	48	1.2	0.025	NR	FT‐IR	[29]
*Halomonas smyrnensis* AAD6T	Sucrose	20	Batch	Bioreactor	24	18.06	0.106	NR	HPLC	[30]
			Fed‐batch	Bioreactor		12.20	0.074			

*B. subtilis* FCBP‐SB‐0189	Mango peel hydrolysate	50	Batch (SmF)	Shake flask (250 mL)	64	0.717	0.011	NR	HPLC	[31]
*Azotobacter chroococcum* DSOSMOTICM2286	Sucrose	419	Batch	Shake flask	48	387.4	8.07	NR	HPLC	[32]
*Leuconostoc citreum* BD1707	Sucrose	172	Batch	Shake flask	112	34.86	0.31	NR	HPLC	[33]
*B. subtilis* MT453867	Sucrose	80	Batch	Shake flask	54	33	0.61	NR	TLC, FT‐IR and NMR	[34]
*Paenibacillus polymyxa*	Sucrose	125	Batch	Shake flask	96	68	0.71	NR	FT‐IR and NMR	[35]
*Gluconobacter japonicus* LMG1417	Sucrose	720	Cell‐free enzymatic production	Culture supernatant reaction	24	157.9	6.58	NR	HPLC	[36]
*Tanticharoenia sakaeratensis*	Sucrose	200	Batch	Shake flask	35	24.7	0.71	NR	FT‐IR and NMR	[37]
*B, subtilis* M	Sucrose	100	Batch	Bioreactor 150 L	24	47	1.96	29.4	FT‐IR	[38]
*Saccharomyces cerevisiae*	Sucrose	191	Fed‐batch	Bioreactor 50 L	24	76	3.17	0.8	HPLC	[39]

*Note:* Levan production under different cultivation conditions: Compilation of levan production by different microorganisms under diverse fermentation conditions, including substrate type, sucrose concentration, fermentation mode, reactor scale, cultivation time, productivity, and analytical methods. Due to heterogeneous experimental conditions (e.g., shake flasks vs. bioreactors, different fermentation times and methods). Average productivity values (g L^−1^ h^−1^) were calculated from reported levan concentration and cultivation time when not explicitly provided in the original studies. Reported substrate concentrations correspond to initial or feed concentrations as described in the original studies. For *G. japonicus* LMG1417, levan production corresponded to a cell‐free enzymatic system using culture supernatant containing secreted levansucrase rather than direct microbial fermentation.

Abbreviations: Conc., levan concentration; NR, not reported; Productivity (g L^−1^ h^−1^), volumetric levan production rate; SmF, submerged fermentation; Yield (g g^−1^), grams of levan produced per gram of sucrose consumed; Yp/s, g levan/g substrate; Yp/x, g levan/g biomass.

The data summarized in Table 1 highlight substantial variability in reported levan production values (<1 to >700 g L^−1^), reflecting not only microbial diversity but also major differences in process configuration, substrate strategy, reactor operation, and analytical methods. Consequently, these values should be interpreted cautiously rather than directly compared, as the underlying systems differ substantially in reactor configuration, mixing and oxygen transfer capacity (kLa), aeration, pH control, cultivation strategy, and quantification methodology. These limitations underscore the need for a structured and process‐oriented analysis of the key factors governing levan production, including operational constraints, substrate effects, and challenges associated with performance normalization.

#### 3.1.1. Operational Constraints and Reactor‐Dependent Variability in Levan Production

Levan production has been investigated across a wide range of experimental systems, from shake flasks to controlled bioreactors. Laboratory‐scale studies report, for instance, 34.86 g L^−1^ for *L. citreum* after 112 h [33] and 68 g L^−1^ for *P. polymyxa* after 96 h [35], whereas larger‐scale processes achieved 47 g L^−1^ for *B. subtilis* in a 150 L bioreactor under batch conditions [38] and 76 g L^−1^ for *S. cerevisiae* in a 50 L fed‐batch system [39]. Consequently, the reported levan concentrations reflect not only microbial performance but also differences in bioreactor design and operational conditions. This limitation is further reinforced by the inconsistent reporting of yield coefficients (g g^−1^), which are available only in a subset of studies (e.g., 0.28 and 0.45 [28], 0.93 [19], and 0.8 [39]) and are often ambiguously defined (initial vs. consumed substrate), thereby restricting robust cross‐study comparisons. Among process parameters, oxygen transfer and mixing efficiency appear to be key constraints. Oxygen availability, commonly described by the volumetric oxygen transfer coefficient (kLa), has been identified as a critical factor in aerobic and facultative systems such as *Bacillus*, *Azotobacter*, and *Gluconobacter.* In addition, high sucrose concentrations (>500 g L^−1^) may increase medium viscosity, thereby limiting mass and oxygen transfer efficiency, as reported in high‐substrate fermentation systems [19, 36]. From a physiological perspective, these constraints appear to be strain‐dependent. Strictly aerobic bacteria such as *Gluconobacter* spp. rely on oxygen‐dependent metabolism, whereas facultative anaerobes such as *Bacillus spp.* can adapt to oxygen limitation by switching to alternative metabolic pathways [40, 41]. Oxygen availability has been reported to influence cellular redox balance and metabolic flux distribution, thereby affecting microbial production yields and potentially influencing levansucrase activity and levan characteristics, depending on the producing strain and cultivation conditions [42].

Case studies further illustrate the complexity of process interpretation. For example, *H. smyrnensis* [30] showed higher performance in batch than in fed‐batch conditions; however, these differences cannot be attributed solely to feeding strategy, as reactor configuration and oxygen transfer likely play major roles. Similarly, recombinant *P. pastoris* [28] exhibited significantly higher titers and yields in a controlled bioreactor (72.9 g L^−1^; 1.24 g L^−1^ h^−1^; 0.45 g g^−1^) compared with shake flasks (8.62 g L^−1^; 0.03 g L^−1^ h^−1^; 0.28 g g^−1^), highlighting the importance of process control.

Overall, these observations demonstrate that levan production performance is strongly constrained by reactor‐dependent factors and poorly standardized metrics, limiting meaningful comparisons across studies. This highlights the need for harmonized reporting (e.g., kLa, yield definitions, and productivity metrics) and for process‐oriented analyses that explicitly integrate oxygen transfer, mixing, and substrate utilization to enable rational scale‐up and robust bioprocess design.

#### 3.1.2. Substrate‐Driven Effects: Concentration, Feedstock Variability, and Process Limitations

Sucrose remains the primary substrate due to levansucrase specificity; however, both substrate type and concentration strongly influence production outcomes. Reported initial sucrose concentrations vary widely, ranging from 20 g L^−1^ (*H. smyrnensis*) [30] to 720 g L^−1^ (*G. japonicus*) [36]. Although high levan titers are frequently reported at elevated sucrose concentrations, no universal relationship can be established. Alternative substrates introduce significant variability in levan production, affecting both titers and production kinetics. Sugarcane molasses enabled rapid synthesis by *S. kilbournensis* (44.33 g L^−1^ in 6 h; 7.39 g L^−1^ h^−1^) [12], whereas mango peel hydrolysate used with *B. subtilis* FCBP‐SB‐0189 yielded only 0.717 g L^−1^ after 64 h [31]. These differences reflect variations in sugar composition, carbon accessibility, and the presence of inhibitory compounds. Although yield coefficients are reported for sucrose‐based systems (up to ~0.93 g g^−1^ [19]), they remain unavailable for most alternative substrates, preventing rigorous comparison of substrate efficiency across feedstocks. As reported by Shang et al., increasing sucrose concentration enhances levan production up to an optimal threshold, beyond which process performance declines due to viscosity‐related effects [43].

Building on these findings, very high sucrose concentrations introduce significant process constraints. Increased viscosity reduces mixing and mass transfer efficiency, whereas the limited availability and heterogeneity of yield data further complicate evaluation of substrate utilization efficiency. These conditions likely reflect optimized laboratory‐scale systems rather than industrially scalable processes.

#### 3.1.3. Limitations of Performance Metrics: Challenges in Normalization and Cross‐Study Comparison

To partially account for differences in cultivation time, volumetric productivity (g L^−1^ h^−1^) is often considered a more informative metric than concentration alone. High productivities have been reported for *A. chroococcum* (8.07 g L^−1^ h^−1^) [32], *G. japonicus* (6.58 g L^−1^ h^−1^) [36], and *S. kilbournensis* (7.39 g L^−1^ h^−1^) [12]. However, productivity alone does not fully capture process efficiency. Yield coefficients (g g^−1^) are reported in a subset of studies, ranging from approximately 0.28–0.93 [19, 28]; however, their inconsistent availability and lack of standardization limit their use as a universal comparison metrics. In some cases, reported values appear unusually high (e.g., >1 g g^−1^). The reported value of 29.4 g g^−1^ corresponds to the specific yield (Yp/x), reflecting levan production per unit of biomass rather than substrate conversion efficiency [38]. From a bioengineering perspective, these limitations may restrict the accurate evaluation of carbon partitioning and metabolic efficiency across systems. Analytical heterogeneity further complicates comparisons, as methods such as HPLC, FT‐IR, and NMR differ in quantification accuracy, and discrepancies may arise depending on whether levan is quantified directly in the broth or after purification [44]. Notably, reported yield values can vary substantially across systems. For example, recombinant *P. pastoris* exhibited higher yields under controlled bioreactor conditions (0.45 g g^−1^) compared with shake flasks (0.28 g g^−1^) [28], suggesting that process control may influence carbon conversion efficiency.

To address these limitations, future studies should adopt standardized and clearly defined performance metrics, including yield based on consumed substrate (g g^−1^), volumetric productivity (g L^−1^ h^−1^), and, where possible, specific productivity (g g^−1^ biomass h^−1^). In addition, harmonized analytical methods and explicit reporting of quantification procedures (e.g., crude vs. purified levan) would improve data consistency. Such standardization would enable more reliable cross‐study comparisons and support the development of quantitatively robust bioengineering models.

#### 3.1.4. Bioprocess Design as the Primary Driver of Levan Production Performance

This section provides a process‐oriented synthesis of the available data, emphasizing that levan production performance is primarily governed by bioprocess design and mass transfer constraints. Comparative analyses indicate that parameters such as substrate strategy, oxygen transfer capacity (kLa), and cultivation mode collectively shape production outcomes, although their relative contributions remain difficult to quantify due to inconsistent experimental designs and reporting practices. For instance, recombinant *P. pastoris* exhibits substantially higher titers and yields under controlled bioreactor conditions than in shake flasks [28], whereas *H. smyrnensis* shows variable performance between batch and fed‐batch systems depending on operating conditions [30]. Similarly, the very high titers reported for *B. velezensis* under continuous operation appear to be strongly dependent on process configuration [19]. These observations suggest that high volumetric production does not necessarily reflect efficient substrate conversion, but may instead result from optimized reactor conditions. In this context, oxygen availability, commonly described by the volumetric oxygen transfer coefficient (kLa), appears to play a central role. As reflected in Table 1, strictly aerobic microorganisms such as *G. japonicus* [36] and *A. chroococcum* [32] tend to exhibit high productivity under conditions of efficient oxygen transfer, whereas facultative anaerobes such as *B. subtilis* [27, 38] and *P. polymyxa* [27] may tolerate a broader range of oxygen conditions. However, in high‐viscosity systems associated with elevated sucrose concentrations, oxygen transfer limitations may further constrain process efficiency.

Overall, the available data remain limited by insufficient standardization of experimental conditions and normalized performance metrics, which restricts robust cross‐study comparisons. These limitations suggest that apparent trends should be interpreted with caution. Future progress will require harmonized reporting practices and process‐integrated analyses that explicitly consider mass transfer (kLa), substrate utilization efficiency, and reactor design.

This framework emphasizes that process selection must be application‐driven, requiring alignment between performance targets (yield vs. productivity), scalability constraints, and key engineering parameters such as feeding strategy and oxygen transfer. Rather than identifying a universally superior cultivation mode, it provides a rational basis for selecting the most suitable process configuration according to industrial objectives.

### 3.2. Diversity of Levansucrase‐Encoding Genes

The variability in levan yield and structural properties among microbial producers largely arises from the molecular diversity and regulatory complexity of levansucrase‐encoding genes. Understanding enzyme function and gene regulation is therefore essential for optimizing levan biosynthesis. Levansucrase, invertase, and fructosyltransferases are key enzymes involved in fructan metabolism [45]. Fructosyltransferases exhibit both hydrolytic and transfructosylating activities, enabling sucrose cleavage and fructosyl transfer to acceptor molecules, leading to the formation of fructooligosaccharides or polymeric fructans [22]. Levansucrase catalyzes both sucrose hydrolysis and fructosyl transfer during levan synthesis, whereas invertase primarily hydrolyzes sucrose into glucose and fructose, contributing to cellular metabolism and potentially competing with levan formation [46]. Levansucrase then catalyzes the transfer of fructosyl units to elongating fructan chains, forming *β*‐(2 → 6)‐glycosidic linkages characteristic of levan [47]. Both enzyme activity and environmental conditions govern polymer yield and molecular weight (MW) distribution. At the molecular level, enzyme concentration plays a critical role in polymer architecture. Raga‐Carbajal et al. [48] demonstrated in *B. subtilis* that low SacB levels favor processive polymerization, leading to high–molecular‐weight levan, whereas high enzyme concentrations promote nonprocessive elongation and shorter chains. Genetic organization further modulates this process. In *B. subtilis*, levan biosynthesis is controlled by the sacB–levB–yveA tricistronic operon. SacB encodes levansucrase, whereas levB encodes an endolevanase that hydrolyzes high–molecular‐weight levan into smaller oligosaccharides. Importantly, levB also enhances sacB transcription via a positive regulatory mechanism, forming a feedback loop that links polymer synthesis and degradation. The role of yveA remains unclear but has been suggested to involve transport functions [4]. This regulatory mechanism is further reinforced by SacY‐mediated antitermination. Under sucrose‐rich conditions, SacY binds to the ribonucleic antiterminator (RAT) sequence within the *sacB* transcript, preventing premature transcription termination and thereby enhancing levansucrase expression and levan production, as demonstrated by Tortosa et al. [49]. This mechanism is well established and has been consistently supported by subsequent studies on RNA‐binding antiterminators, which confirm that SacY controls *sacB* expression through sucrose‐responsive transcriptional regulation. Extremophilic levansucrases, such as those from halophilic microorganisms, exhibit sequence adaptations that enhance catalytic stability under harsh environmental conditions. According to Kırtel et al. [30], the levansucrase from *H. smyrnensis* demonstrates remarkable activity and stability at high salt concentrations, highlighting its adaptation to saline environments. These characteristics support efficient levan synthesis under extreme conditions and emphasize the potential of extremophilic enzymes for industrial applications. According to Xu et al. [45], variations in gene sequences directly influence enzyme structure, catalytic activity, and product specificity, thereby determining the MW and architecture of the synthesized levan. Beyond operon regulation, enzyme localization, gene copy number, and host system significantly influence enzyme availability and secretion efficiency, ultimately affecting polymer yield and structure, as demonstrated in heterologous systems such as *S. cerevisiae* [17]. Furthermore, sequence adaptations in extremophilic levansucrases and targeted mutations, such as S164A in *B. subtilis*, have been shown to shift the balance between hydrolytic and transfructosylation activities, thereby modulating levan MW distribution [50].

**Figure 2:**
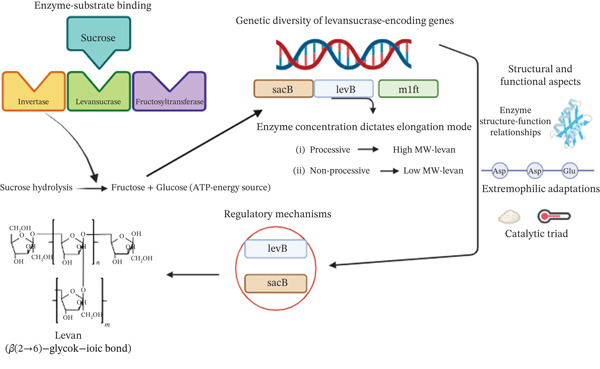
Schematic representation of levan biosynthesis and its genetic regulation. Sucrose is hydrolyzed by invertase, releasing glucose and fructose. The glucose produced is utilized as an energy source through central metabolic pathways, whereas fructose serves as the substrate for levan synthesis. Fructose units are subsequently polymerized by levansucrase and fructosyltransferases into levan, a *β*‐(2 → 6)‐linked fructose polymer. Genes such as *sacB*, *levB*, and *m1ft* (encoding fructosyltransferases) regulate enzyme expression, whereas structural features, including the Asp–Asp–Glu catalytic triad essential for fructosyl transfer and extremophilic adaptations, govern enzyme activity and stability.

Based on the proposed regulatory scheme (Figure 2), several studies demonstrate a clear relationship between genetic organization, enzyme activity, and levan MW. The *sacB-levB-*yveA operon plays a central role in this process, where levansucrase (*sacB*, GH68) catalyzes sucrose hydrolysis and fructosyl transfer, whereas endo‐levanase (*levB*, GH32) modulates polymer size through partial hydrolysis. Reduced expression or absence of *levB* promotes the accumulation of high‐molecular‐weight (HMW) levan, whereas its activity favors the formation of shorter fructooligosaccharides. In addition, SacY‐mediated antitermination enhances *sacB* expression under sucrose‐rich conditions via a feedback mechanism involving *levB*, thereby increasing levan production [51].

Overall, the figure highlights that levan yield and MW are tightly regulated by the interplay between genetic factors, enzyme activity, and process conditions. As supported by González‐Garcinuño et al., sucrose concentration and microbial characteristics directly influence levansucrase behavior, thereby controlling polymer elongation and accumulation [26]. This integrated network ultimately determines whether conditions favor HMW levan with high yield or lead to reduced polymer size and lower productivity.

**Figure 3:**
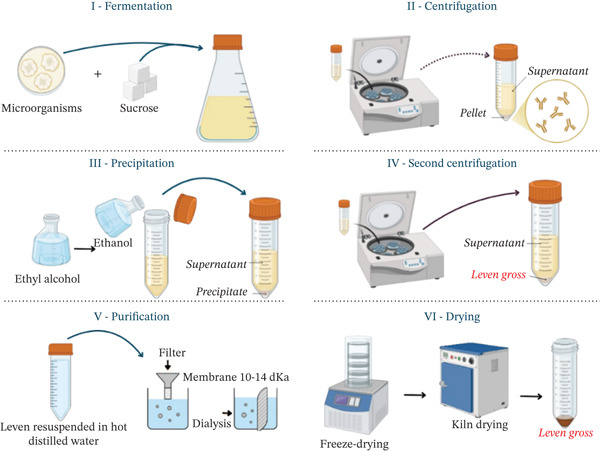
Main steps involved in levan purification and recovery. Following microbial fermentation, biomass is removed by centrifugation, after which levan is recovered through ethanol‐induced precipitation and secondary centrifugation. Subsequent purification steps, including filtration, dialysis, or ultrafiltration, may be applied prior to final drying to obtain high‐purity levan.

Table 2 provides a comparative overview of selected studies highlighting how different genetic configurations, expression systems, and process conditions influence levansucrase activity and levan production. Unlike the conceptual framework presented in Figure 3, this table emphasizes experimentally validated strategies and their specific impact on polymer yield, MW, and process performance.

**Table 2:** Genetic and enzymatic determinants governing levan biosynthesis and polymer architecture in microbial systems.

Microorganisms	Gene(s)/enzyme(s)	Regulatory and enzymatic mechanisms	Key findings	Bioengineering implications	References
*Bacillus subtilis*	sacB	Levansucrase expression; enzyme concentration‐dependent activity	Enzyme concentration (not substrate) determines elongation mode: ‐low enzyme concentration → processive synthesis of HMW levan ‐High enzyme concentration → nonprocessive synthesis of LMW levan	Molecular weight can be tuned by controlling enzyme concentration rather than substrate concentration	[52]
*B. subtilis*	sacB, levB	Nonprocessive fructosyl transfer network; relaxed acceptor specificity	Nonprocessive synthesis produces >150 intermediates (DP up to ~70) due to broad acceptor specificity, generating diverse LMW levan fractions and oligosaccharides	Controlling acceptor reactions and intermediates is key to tailoring LMW levan and oligosaccharide profiles	[48]
*B. subtilis*	sacB–levB–yveA	SacY − dependent antitermination + levB transcript − mediated feedback	Via a SacY‐dependent regulatory mechanism; increasing operon expression under sucrose conditions	Regulatory RNA‐based feedback can be exploited to boost levansucrase expression independently of enzymatic activity	[51]
*B. subtilis (*S164A levansucrase mutant)	sacB (mutant)	Altered catalytic balance (transfructosylation vs. hydrolysis)	Mutation modifies transfructosylation/hydrolysis balance, affecting polymer size distribution	Enzyme engineering enables control of polymer properties via catalytic specificity	[50]
*Saccharomyces cerevisiae* (heterologous)	m1ft (*Leuconostoc mesenteroides*)	Heterologous expression; secretion/localization effects	Functional levan synthesis achieved; intracellular accumulation enhanced without signal peptide; coexpression with sucrose transporter (SUT) enables extracellular production	Efficient production depends on sucrose availability, transport, and secretion; competition for sucrose‐derived carbon flux is a key limitation	[17]
*Azotobacter spp.*	levB (LevB2286)	Endo‐levanase hydrolysis of levan	Combined with levansucrase activity, enables efficient conversion of sucrose into FOS‐rich products	Coupling polymer synthesis and controlled hydrolysis enables high‐yield production of tailored prebiotic oligosaccharides	[32]
*Gluconobacter spp.*	levS1417 (levansucrase)	High‐activity enzyme secretion; cell‐free catalysis	Extremely high enzyme activity (>4500 U/mg); high levan yield (157.9 g/L) in cell‐free system; overexpression significantly improves space–time yield; minimal product inhibition	Cell‐free production using supernatant + enzyme overexpression enables very high productivity and scalable processes	[36]

*Note:* Bioengineering insights into microbial levan production: linking enzymatic mechanisms to process‐relevant outcomes. Overview of key microorganisms, genes, and enzymatic mechanisms involved in microbial levan biosynthesis, with experimentally validated findings and their associated bioengineering implications. The table highlights how molecular‐level determinants (e.g., enzyme concentration, catalytic specificity, regulatory RNA mechanisms, and secretion efficiency) directly influence polymer characteristics and process performance.

Abbreviations: DP, degree of polymerization; FOS, fructooligosaccharides; g L^−1^, grams per liter; HMW, high molecular weight; LevB, endo‐levanase; LevS1417, levansucrase from *Gluconobacter japonicus* LMG 1417; LMW, low molecular weight; SUT, sucrose transporter; SacB, levansucrase from *Bacillus subtilis*; U mg^−1^, enzyme activity units per milligram of protein.

Across microbial systems, levansucrase functionality emerges as a multifactorial trait governed by enzyme concentration, catalytic specificity, regulatory mechanisms, and host‐dependent constraints. In native producers such as *B. subtilis*, recent mechanistic studies indicate that polymer MW is not primarily controlled by substrate concentration but by enzyme concentration, which dictates the elongation mechanism. Specifically, low enzyme concentration favors a processive mechanism leading to HMW levan, whereas high enzyme concentration promotes a nonprocessive mechanism generating low‐molecular‐weight (LMW) levan through a broad spectrum of intermediates [52]. This nonprocessive synthesis is characterized by relaxed acceptor specificity and the formation of more than 150 intermediate oligosaccharides (DP up to ~70), highlighting the complexity of the underlying fructosyl transfer network [48].

In addition to enzymatic control, operon‐level regulation plays a critical role. In *B. subtilis*, expression of the sacB–levB–yveA operon is regulated by a SacY‐dependent antitermination mechanism, further modulated by a positive feedback loop mediated by the levB transcript, independently of LevB enzymatic activity [51]. This RNA‐based regulatory mechanism enhances sacB transcription under sucrose conditions, providing a molecular basis for sustained levansucrase production that is distinct from purely metabolic control.

Enzyme engineering further expands the control space. For example, site‐directed mutations in levansucrase (e.g., S164A) alter the balance between transfructosylation and hydrolysis, thereby modifying polymer size distribution [50]. Such findings confirm that catalytic specificity is a key determinant of polymer architecture and can be rationally tuned.

In heterologous systems such as *S. cerevisiae* expressing the M1FT levansucrase from *L. mesenteroides*, functional levan synthesis has been demonstrated, with production strongly influenced by intracellular sucrose availability, enzyme localization, and secretion efficiency [17]. Notably, intracellular accumulation is enhanced in the absence of a signal peptide, whereas coexpression with a sucrose transporter enables extracellular production, underscoring the importance of transport and metabolic flux distribution.

Beyond polymer synthesis, downstream enzymatic modification also plays a crucial role in product diversification. For instance, the endo‐levanase LevB2286 from *Azotobacter* spp. enables controlled hydrolysis of levan into fructooligosaccharides (FOS, DP2–DP8) with minimal fructose release [32]. When combined with levansucrase activity, this system allows efficient one‐step conversion of sucrose into prebiotic oligosaccharides at high titers (~387 g L^−1^). Similarly, in *Gluconobacter* spp., the secretion of a highly active levansucrase (LevS1417) supports high‐yield levan production in cell‐free systems (≈157.9 g L^−1^), with enzyme overexpression significantly improving space–time yields and exhibiting minimal product inhibition [36].

Overall, these findings move beyond descriptive insights into levansucrase genetics and reveal a set of actionable engineering levers. In particular, enzyme concentration, catalytic specificity, and regulatory RNA mechanisms emerge as primary determinants of polymer MW and yield, whereas host‐dependent factors such as secretion efficiency and substrate accessibility define process performance. This integrated perspective highlights the importance of linking genetic regulation, enzyme behavior, and process configuration to enable the rational design of levan production systems with tailored structural and functional properties.

### 3.3. Levan Purification

Beyond production, downstream processing is a critical step that determines levan purity, molecular characteristics, and industrial applicability across food, pharmaceutical, and cosmetic sectors. Although laboratory‐scale purification is relatively straightforward, scale‐up remains constrained by significant downstream bottlenecks. Conventional workflows typically involve biomass removal by centrifugation, followed by ethanol precipitation to recover crude levan, a second centrifugation step, and subsequent polishing operations such as filtration, dialysis, or ultrafiltration prior to drying [53, 54]. However, these multistep strategies may present substantial technical and economic limitations at industrial scale. In particular, the large volumes of solvent required for precipitation, the limited scalability of dialysis, and the complexity of sequential purification steps collectively represent major constraints. As reported by Dahech et al. [55], these limitations can hinder process scalability and increase operational costs. In addition, drying remains challenging: although lyophilization is commonly used at laboratory scale, it is difficult to scale, whereas spray drying may require carriers due to the adhesive nature of levan, potentially reducing product purity [20].

Centrifugation plays a key role in solid–liquid separation; however, its efficiency may be influenced by broth properties. High sucrose concentrations, often used to enhance levan production, can increase medium viscosity, which may reduce sedimentation efficiency and impair phase separation. Consistent with the observations of Mottola et al. [56], levan recovery typically involves solvent‐intensive precipitation (e.g., ethanol addition in large excess), followed by low‐temperature incubation, centrifugation, and freeze‐drying, highlighting the operational complexity and limited scalability of current purification workflows. Ethanol precipitation is widely used for levan recovery based on solubility differences, but efficient purification often requires additional steps such as salt precipitation and chromatographic techniques. As shown by Chaudhary et al. [57], these multistep strategies are essential to improve purity and remove coextracted impurities. However, these approaches are associated with high costs related to solvent consumption, recovery, and safety management, as well as the capital and energy demands of separation and purification equipment.

Importantly, downstream efficiency is strongly coupled with upstream process constraints. High sucrose concentrations increase broth viscosity, thereby limiting mass transfer, reducing sedimentation rates, and impairing centrifugation efficiency. Consistent with the findings of Ren et al. [58], system performance is also influenced by microbial aggregation and structural organization, which can either enhance or hinder transport phenomena depending on operating conditions. These results indicate that separation efficiency is governed by the combined effects of fluid properties and biomass organization.

In addition, ethanol‐based precipitation, whereas effective at laboratory scale, raises significant concerns regarding solvent consumption, recovery, and environmental impact at industrial scale. Wang and Dennis demonstrated that optimized process design can significantly improve energy efficiency and reduce environmental impact. For instance, advances in engineered systems have demonstrated substantial gains in performance (e.g., increased efficiency and reduced emissions) through improved process configurations [59]. More broadly, downstream processing is frequently identified as a major contributor to the overall economic and environmental footprint of polysaccharide production. Studies on bioprocess systems suggest that energy‐ and solvent‐intensive operations may dominate process costs and environmental impacts, emphasizing the need for improved process integration and optimization. Overall, downstream processing remains a major bottleneck due to its high energy, solvent demand, and limited scalability, highlighting the need for integrated and more sustainable purification strategies.

Figure 3 summarizes the main purification steps for levan recovery, illustrating the typical downstream process after microbial fermentation, centrifugation, ethanol‐induced precipitation, and MW fractionation commonly used for its simplicity and effectiveness in producing purified levan for diverse applications.

### 3.4. Sustainability Assessment Framework for Levan Bioprocesses

The increasing interest in levan as a bio‐based polymer necessitates the integration of sustainability metrics into process design and evaluation. Although most studies focus on production yield and productivity, the environmental and economic impacts of levan bioprocesses remain insufficiently addressed. As highlighted by Fu et al., techno‐economic analysis (TEA) and life cycle assessment (LCA) are essential complementary tools for evaluating economic feasibility and environmental sustainability, as well as for identifying process bottlenecks and guiding optimization strategies [60]. A structured sustainability assessment framework integrating LCA and TEA is increasingly recognized as essential for evaluating the environmental and economic performance of bioprocesses. As highlighted by Ferdous et al., recent developments emphasize integrated approaches combining LCA and TEA with process simulation and multiobjective optimization, enabling a more robust and holistic evaluation of technical, economic, and environmental trade‐offs [61]. LCA provides a systematic approach to quantify environmental impacts across the entire production chain, from raw material extraction to product recovery, following a cradle‐to‐gate perspective. As demonstrated by Wan Jo et al., such analyses emphasize key indicators including global warming potential and energy consumption, showing that reducing process temperature and energy demand can significantly lower CO2 emissions and improve overall sustainability performance [62].

In fermentation‐based systems, both upstream processes and downstream operations may contribute significantly to the overall environmental burden. Although upstream optimization can reduce energy demand, these gains may be partially offset by energy‐ and solvent‐intensive downstream steps. The findings of Kuntal Jana and Sudipta De [63] further suggest that specific process stages may dominate overall environmental impacts depending on system configuration and energy requirements. Consistent with this, downstream processing is often considered a major environmental hotspot, particularly due to solvent‐based recovery and drying operations [63]. This highlights the need for integrated optimization of both upstream and downstream stages to achieve sustainable bioprocess design.

Based on these considerations, the sustainability of levan production can be interpreted through four interconnected dimensions, as reflected in the variability observed in Table 1.

The first dimension relates to substrate sustainability. The use of low‐cost feedstocks such as molasses (e.g., *S. kilbournensis*, 44.33 g L^−1^ in 6 h [12]) is associated with high productivity, whereas lignocellulosic hydrolysates (e.g., mango peel, 0.717 g L^−1^ [31]) show lower efficiency, highlighting a trade‐off between cost and performance. The second dimension concerns process efficiency. Reported yields and productivities vary widely (e.g., Yp/s from 0.28 to 0.93 in *P. pastoris* and *B. velezensis*), suggesting that high titers (e.g., 746 g L^−1^ in *B. velezensis*) do not necessarily correspond to optimal substrate conversion [19, 28]. The third dimension involves energy demand and oxygen transfer. High sucrose concentrations (up to 720–800 g L^−1^) may enhance production but also increase broth viscosity, which could limit mass and oxygen transfer and increase energy requirements, thereby constraining scalability [19, 36]. The fourth dimension focuses on downstream processing. Recovery efficiency and purification strategy strongly influence overall process performance. Typical operations include centrifugation (8000–12,000 × g, 10–20 min, 4°C) and ethanol precipitation (1:1–3:1), which remain widely used but are energy‐ and solvent‐intensive [30].

In addition, ethanol precipitation combined with dialysis, although effective at laboratory scale, may be difficult to scale due to high solvent consumption and long processing times [64].

These limitations may be exacerbated when processing high‐viscosity broths, where separation efficiency could decrease, leading to increased energy demand and operational costs. Consequently, growing attention has been directed toward alternative downstream strategies. Membrane‐based processes, particularly ultrafiltration, offer selective and scalable solutions for concentration and purification, whereas microfiltration may be required for complete biomass removal [65–67].

Overall, these constraints, together with the variability highlighted in Table 1, underscore the need for integrated process design that simultaneously considers upstream conditions and downstream efficiency. In this context, process intensification combined with alternative strategies such as membrane filtration, continuous clarification, and flocculation‐assisted recovery may represent a promising pathway toward sustainable and scalable levan bioprocesses.

### 3.5. Application Driven Design of Levan Bioprocesses: Linking Structure, Function, and Process Parameters

The functional properties of levan depend strongly on its MW, degree of branching, and purity, which are governed by the interplay between microbial diversity, levansucrase regulation (Section 3.2), fermentation conditions (Section 3.1), and downstream processing (Section 3.3). These factors collectively define polymer structure and, consequently, application performance, highlighting the need to integrate biological and engineering parameters in process design [30].

In food systems, levan is mainly used as a stabilizer, thickener, and fat replacer [68]. These functionalities are generally associated with HMW polymers that enhance viscosity and water retention. Such structures may be favored at high sucrose concentrations, which promote polymer elongation [69]. However, as discussed in Section 3.1, these conditions may also increase broth viscosity and limit oxygen transfer (kLa), thereby constraining scalability [70]. From a downstream perspective, food applications generally tolerate moderate purity levels; therefore, crude or partially purified levan may be acceptable depending on formulation requirements and regulatory constraints. This enables simpler recovery processes such as ethanol precipitation, reducing cost and energy demand. Several studies have validated levan functionality in real food systems (e.g., yogurt), indicating a relatively high technological readiness level [71]. These considerations suggest that optimizing the trade‐off between high MW and process operability remains essential, requiring improved control of sucrose feeding, viscosity, and oxygen transfer to ensure scalable food‐grade levan production.

In contrast, pharmaceutical and medical applications typically require strict control of MW distribution, low polydispersity, minimal endotoxin and protein content, as well as controlled viscosity, as reported by Srikanth [72]. These requirements highlight the need for tight regulation of levansucrase activity, involving controlled expression of *sacB* and modulation of levan‐degrading enzymes such as *levB*, in accordance with the desired polymer size [73]. Downstream processing becomes a major constraint, as ultrafiltration, sterile filtration, and chromatographic steps are often required to achieve pharmaceutical‐grade purity, thereby increasing both cost and environmental impact. In this context, Emami et al. demonstrated that ultrafiltration of polysaccharides is strongly limited by concentration polarization and membrane fouling, which reduce transmission efficiency and necessitate high diafiltration volumes, further increasing process complexity and energy demand [74]. In addition, levan has emerged as a promising biomaterial for medical applications due to its antimicrobial and immunomodulatory [75] and regenerative properties [76]. It has demonstrated significant potential in anticancer, antiparasitic, antioxidant [77], and cholesterol‐lowering (LDL) activities [78]. However, such applications require strict control of MW distribution, low polydispersity, and high purity. This necessitates tight regulation of levansucrase activity, including controlled expression of *sacB* and modulation of *levB*, as well as intensive downstream processing steps, which increase both process complexity and cost.

Cosmetic applications represent an intermediate case, requiring moderate MW (LMW fractions) and relatively low impurity levels [4]. Levan has been shown to contribute to the stabilization, thickening, and other functional properties of cosmetic formulations [79]. Partial purification strategies are generally sufficient, reducing downstream intensity compared with pharmaceutical applications. However, variability in polymer structure resulting from fluctuations in fermentation conditions such as substrate composition and oxygen transfer may affect formulation reproducibility [20]. Modified levan derivatives have shown potential to enhance biological responses such as skin regeneration [80]. The work of Pérez‐Rivero and López‐Gómez highlighted the pivotal role of biotechnology, particularly fermentation‐based processes, in the development of sustainable cosmetic ingredients. Their study demonstrated that fermentation enables the production of bio‐based, multifunctional compounds with enhanced biocompatibility, biodegradability, and reduced environmental impact compared with conventional synthetic ingredients. Furthermore, bioprocessing facilitates the transformation of substrates into bioactive metabolites with improved functionality and bioavailability, which is highly relevant for skin applications. Importantly, the authors emphasized that downstream processing, including purification and standardization, remains essential to ensure the safety, stability, and reproducibility of cosmetic formulations, despite generally less stringent requirements than in pharmaceutical applications [81].

From a bioengineering perspective, application‐specific requirements reveal key limitations in current levan production systems. HMW levan for food applications may be achieved under elevated sucrose concentrations; however, increased broth viscosity can limit mass and oxygen transfer, thereby constraining scalability. This suggests the need for optimized feeding strategies and improved oxygen transfer capacity (kLa). In pharmaceutical and medicine applications, strict control of MW and purity requires precise regulation of levansucrase activity, including control of sacB expression and modulation of levan‐degrading enzymes such as levB. However, this increases process complexity and downstream costs.

**Figure 4:**
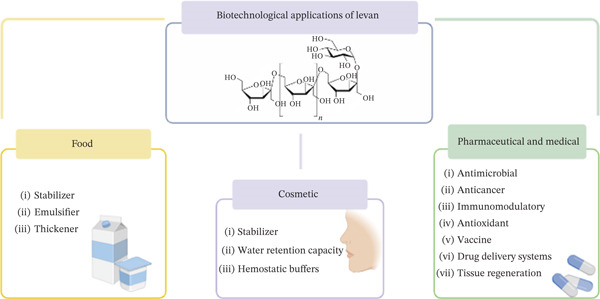
Schematic overview of the main biotechnological applications of levan. The figure illustrates the multifunctional roles of levan across the food, cosmetic, pharmaceutical, and biomedical sectors, highlighting its broad industrial relevance and application potential.

To address these constraints, appropriate process strategies, including batch, fed‐batch, and continuous cultivation, should be considered depending on the desired balance between control, productivity, and scalability. As observed, fed‐batch strategies enable better control of substrate availability and osmotic stress, whereas continuous systems may enhance productivity and process stability. In parallel, advanced and scalable purification methods such as membrane‐based processes should be considered. Cosmetic applications present intermediate constraints, but variability in polymer structure may affect reproducibility, highlighting the need for improved process control and standardization. Across all domains, downstream processing remains a major bottleneck due to membrane fouling and high diafiltration requirements, suggesting that process intensification and alternative recovery strategies, including flocculation‐assisted separation and continuous processing, could improve efficiency. Overall, these limitations underline the need for an integrated bioengineering framework combining genetic, enzymatic, and process‐level optimization to enable scalable and application‐driven levan production. Despite the promising biological properties reported for levan, most pharmaceutical and biomedical applications remain at the in vitro or preclinical stage, with limited evidence currently available from human or clinical studies. Figure 4 synthesizes the key application areas of levan discussed above and illustrates its versatility as a multifunctional biopolymer across diverse industrial sectors.

To synthesize the findings from the reviewed studies, levan production appears to be governed by interconnected mechanisms operating across multiple levels. At the enzyme level, catalytic properties such as processivity and the hydrolysis‐to‐transfructosylation ratio have been reported to influence polymer MW distribution, whereas enzyme concentration and secretion efficiency may affect polymer architecture. At the process scale, factors including high sucrose concentrations (>500 g L^−1^), increased medium viscosity, oxygen transfer limitations (kLa), and osmotic stress have been identified as key constraints that may impact mass transfer, productivity, and scalability.

**Figure 5:**
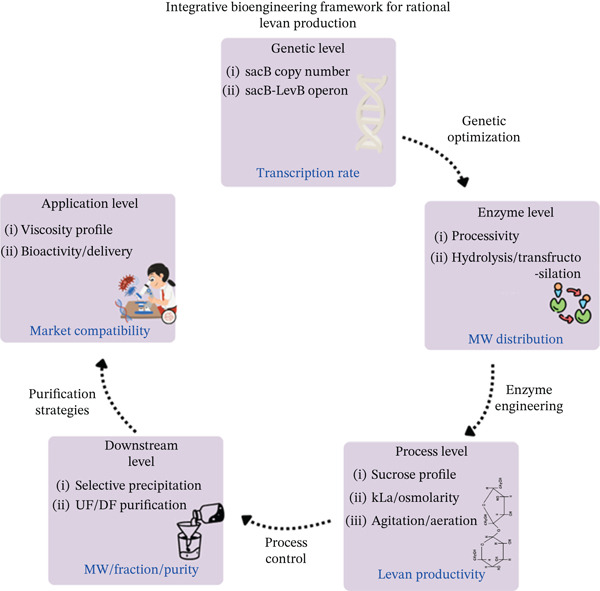
Conceptual bioengineering framework illustrating the multilevel interactions between genetic regulation, enzyme catalytic traits, fermentation parameters, downstream processing strategies, and application requirements in levan production. The framework summarizes the relationships identified across the reviewed studies and highlights how these interconnected factors collectively influence levan yield, molecular weight distribution, purity, and functional properties. Abbreviations: kLa, volumetric oxygen transfer coefficient; UF/DF, ultrafiltration/diafiltration; MW, molecular weight; sacB, levansucrase gene; levB, levanase gene.

Figure 5 integrates these relationships into a conceptual bioengineering framework summarizing the main findings reported in previous studies and reviews, linking genetic regulation, enzyme catalytic traits, fermentation parameters, downstream processing strategies, and application requirements. This framework highlights how these interconnected determinants collectively influence polymer yield, MW distribution, and functional performance, providing a structured basis for rational, application‐driven levan bioprocess design.

Structure function relationships play a central role in determining application suitability. HMW levan is generally associated with enhanced rheological properties in food systems, whereas lower and more defined MW fractions may exhibit improved diffusivity and bioactivity in biomedical applications such as drug delivery or immunomodulation.

Based on these considerations, application‐driven requirements define specific targets in terms of MW, purity, and process design for levan production. Although general trends can be identified, such as high MW for food applications and lower, well‐defined fractions for biomedical uses, these specifications must be balanced with process constraints and scalability. Table 3 summarizes the key design parameters and associated process implications required to achieve fit‐for‐purpose levan across different application sectors, as highlighted in various studies.

**Table 3:** Application‐driven design parameters for rational levan bioengineering.

Application	Target molecular weight	Purity requirement	Key process implications
Food structuring agent	High MW	Food‐grade	High sucrose concentrations; control of viscosity and mass transfer; minimal downstream purification
Beverage texturizer	Moderate‐to‐high MW	Food‐grade	Control of polymer size and rheological properties; optimization of shear and mixing conditions
Drug delivery	Defined low‐to‐moderate MW	Endotoxin‐free; sterile	Controlled enzymatic activity; fed‐batch cultivation strategy; UF/DF purification; sterile filtration
Immunomodulatory applications	Low, well‐defined MW fractions	High purity	Fractionation and molecular weight standardization; advanced purification strategies
Cosmetic formulations	Moderate MW	Low protein content	Controlled precipitation; partial purification; process standardization to ensure reproducibility

*Note:* Application‐driven design parameters for rational levan bioengineering. Overview of target molecular weight (MW) ranges, purity requirements, and key process considerations for levan production across different application sectors, highlighting the relationship between product specifications and bioprocess design.

Abbreviation: UF/DF, ultrafiltration/diafiltration.

Despite these application‐driven trends, levan is frequently characterized by a bimodal MW distribution, comprising both LMW and HMW fractions. This intrinsic heterogeneity arises from the dual catalytic behavior of levansucrase and the influence of reaction conditions on polymerization mechanisms. As a result, the relationship between MW and functional properties cannot be interpreted straightforwardly, as both fractions may coexist and contribute differently to the overall performance. This complexity complicates the establishment of clear structure function correlations and highlights the need for precise control and characterization of levan fractions.

### 3.6. Decision‐Oriented Framework for Application‐Driven Levan Production

To operationalize this conceptual framework within a bioengineering context, levan production can be described as a sequence of interdependent design decisions linking target product properties to process configuration. These decisions involve strain selection, substrate strategy, and process design, which collectively determine product characteristics and process performance.

HMW levan, associated with enhanced rheological and techno‐functional properties, can be produced by various microbial levansucrases via sucrose transfructosylation, generating predominantly linear *β*‐(2,6)‐linked fructan chains that promote HMW fraction formation, as demonstrated by [82], particularly in *Bacillus* systems [68]. In contrast, systems targeting high productivity often rely on strains such as *A. chroococcum* and *G. japonicus*, which have been reported to achieve elevated volumetric productivities, likely due to efficient sucrose metabolism and carbon flux distribution [32, 36]. Additionally, strains such as *Halomonas* spp. may be selected in cases where viscosity control and process robustness are critical.

The second level involves a cost–performance trade‐off driven by substrate selection. Refined sucrose enables controlled kinetics and reproducibility, which may be required for high‐purity or pharmaceutical‐oriented products. In contrast, agro‐industrial substrates such as molasses or hydrolysates can significantly reduce production costs but may introduce compositional variability and potential inhibitors, affecting both yield and reproducibility.

The third level corresponds to process strategy selection, which determines productivity, stability, and scalability. Batch cultivation remains relevant for mechanistic studies and baseline optimization. However, as illustrated in Table 1, fed‐batch systems may allow improved control of substrate availability and osmotic stress, thereby supporting sustained levansucrase activity. Continuous cultivation, when feasible, has been shown to enhance process stability and productivity, as reported for *B. velezensis* systems [19], although operational complexity and contamination risks must be considered.

Across these levels, oxygen transfer (kLa) and mass transfer constraints act as cross‐cutting parameters, particularly at high sucrose concentrations where increased viscosity may limit process performance. These constraints link upstream choices directly to downstream efficiency, as highly viscous broths can reduce separation performance and increase energy demand.

Overall, this review moves beyond a purely descriptive analysis of levan production by establishing a coherent bioengineering framework that links genetic regulation, enzyme functionality, process design, and application‐driven product specifications. By integrating these multilevel determinants, it provides a rational basis for strain selection, process optimization, and downstream processing strategies aligned with targeted MW and purity requirements. Although sustainability considerations are increasingly acknowledged, future research should incorporate quantitative LCA and TEA to enable objective comparison of process configurations. Ultimately, this integrative approach provides a robust foundation for the scalable, sustainable, and application‐oriented development of levan bioprocesses.

## 4. Conclusions

This review highlights that microbial levan production should be interpreted as a multiscale bioprocess resulting from the interplay between genetic regulation, enzyme activity, substrate strategy, and bioprocess conditions. The comparative analysis of the available data indicates that the wide range of reported titers (<1 to >700 g L^−1^) is largely attributable to differences in experimental design and operating conditions rather than intrinsic microbial performance, thereby limiting direct cross‐study comparisons.

The analysis further reveals that the lack of standardized reporting, particularly regarding yield coefficients (g g^−1^ sucrose) and substrate consumption, remains a critical limitation, preventing robust evaluation of carbon conversion efficiency. As a result, high titers or productivities cannot be systematically interpreted as indicators of optimized or scalable processes.

From a bioengineering standpoint, the synthesis of the reviewed studies demonstrates that process design acts as a primary determinant of production performance. In particular, parameters such as substrate concentration, oxygen transfer capacity (kLa), and cultivation mode have been consistently shown to influence metabolic activity, levansucrase expression, and polymer characteristics. Although high sucrose concentrations may enhance polymer synthesis, they also introduce viscosity‐related constraints that can limit mass transfer, process control, and scalability, highlighting inherent trade‐offs between yield and process efficiency.

At the industrial scale, the reviewed studies indicate that downstream processing and sustainability represent major bottlenecks. Conventional purification strategies, especially solvent‐based precipitation, are associated with high‐energy demand and environmental impact, thereby limiting process scalability. These findings emphasize the need for alternative and more efficient separation strategies, including membrane‐based processes, process intensification, and integrated upstream‐downstream optimization.

Beyond these limitations, this review proposes a decision‐oriented bioengineering framework linking genetic determinants, enzyme behavior, process parameters, and downstream strategies to application‐specific requirements. This integrative approach moves beyond descriptive analysis by providing actionable design principles to guide strain selection, process configuration, and product tailoring. Overall, advancing levan bioproduction will require standardized, system‐level approaches integrating performance metrics with sustainability considerations.

## Funding

No funding was received for this manuscript.

## Disclosure

All content was reviewed and edited by the authors, who take full responsibility for the accuracy and integrity of the work.

## Conflicts of Interest

The author declares no conflicts of interest.

## Data Availability

Data sharing is not applicable to this article as no datasets were generated or analyzed during the current study.
